# Thermodynamic simulation on mineralogical composition of CaO–SiO_2_–Al_2_O_3_–MgO quaternary slag system

**DOI:** 10.1186/s40064-016-2289-z

**Published:** 2016-07-08

**Authors:** Chao Liu, Yu-zhu Zhang, Jie Li, Jun-guo Li, Yue Kang

**Affiliations:** School of Materials and Metallurgy, Northeastern University, Shenyang, 110819 China; College of Metallurgy and Energy, North China University of Science and Technology, Tangshan, 063009 China

**Keywords:** FactSage 6.4, Thermodynamic simulation, Mineralogical composition, Quaternary slag system, Hot remelting

## Abstract

It is necessary to elucidate the crystallization thermodynamic of mineralogical phases during the cooling process of the molten BFS with different chemical composition, because the high-melting point mineral phase maybe crystallized during the fiber forming and thereafter cooling process. Thermodynamic calculation software FactSage6.4 and the hot remelting experiments were performed to explore the influence of basicity, Al_2_O_3_ content and MgO content on the crystallization of mineralogical components and their transformation. The results showed that the main mineralography of the CaO–SiO_2_–Al_2_O_3_–MgO quaternary slag system was melilite, and a certain amount of anorthite and calcium metasilicate. The crystallographic temperature of melilite is increased with the increasing of basicity, MgO and Al_2_O_3_ content, which has a significant impact on the utilization performance of the mineral wool prepared with the hot blast furnace slag directly. With the increasing of basicity, there was a tendency that crystallographic amount of melilite increased to the summit and then declined, while the amount of anorthite and calcium metasilicate decreased consistently. Finally, these two mineralogical components could be replaced by magnesium rhodonite and spinel with the increasing of basicity. When the basicity and MgO content were 1.0 and 9 %, the crystallographic mass ratio of melilite and anorthite increased, while that of calcium silicate declined, and replaced by spinel finally with the increasing of Al_2_O_3_ content. When the basicity and Al_2_O_3_ content were 1.0 and 13 %, the crystallographic mass ratio of melilite increased, while that of anorthite and calcium silicate declined, and replaced by pyroxene and spinel with the increasing of MgO content. To decline fiberization temperature of the melt BFS, the basicity, MgO and Al_2_O_3_ content should be decreased during the modification process of chemical composition, because the crystallization temperature of the primary crystalline phase in the slag system without modification.

## Background

Blast furnace slag (BFS) is the main by-product in the process of smelting the pig iron. The quantity of BFS is about 330–450 kg as per ton pig iron produced, which is mainly composed of CaO, SiO_2_, Al_2_O_3_ and MgO, and a kind of non-renewable mineral resources (Yi et al. 2012; Cai et al. 2007). In recent years, the domestic and foreign researchers have gradually realized the broad prospects of valorization of BFS to produce mineral wool, and set out to explore the production technology of mineral wool direct utilizing the molten BFS (Guo et al. 2012; Hua and Fan 2008). The advantages of comprehensive utilization of BFS are energy saving, low cost, and recycling of mineral resource, etc. It has important significance for the iron and steel and mineral industry. Among the mineral wool production methods, melt slag on a spinner wheel is used most commonly. Presently, fiber is formed from the melt film on the spinner wheels is one of the most important but least understood process phenomena. A great amount of input parameters such as rotational frequencies of the spinner wheels, melt string impingement point, melt rheological properties, velocity of the blow-in flow, etc. have significant influence on the fibrosis process. Among these parameters, melt rheological properties were dramatically impacted by the melt temperature and chemical composition. Once the fiberization temperature is low or the chemical composition of melt is unsuitable, the high-melting point mineral phase could be crystallized during the fiberization and thereafter cooling process, which has notably impact on the mechanical property, thermal conductivity, stability and leaching characteristics of the mineral wool. Consequently, application performance of the mineral wool products should be affected. Therefore, it is necessary to elucidate the crystallization thermodynamic of mineralogical phases during the cooling process of the molten BFS with different chemical composition (Wang et al. 2011). In this work, the crystallization thermodynamic of the quaternary slag system of CaO–SiO_2_–Al_2_O_3_–MgO has been simulated with thermodynamics software FactSage 6.4. Simultaneously, the hot remelting test and X-ray analysis have been carried out to determine the mineralogical composition of this quaternary slag system under the temperature of 1250 °C. Then, the crystallization and transformation processes of the high-melting point mineralogical phases such as melilite, dicalcium silicate, anorthite, etc. have been investigated. In view of chemical composition, the influence of basicity, MgO content and Al_2_O_3_ content on crystallization and transformation of these mineralogical phases have been explored. The results obtained in this work could provide experimental and theoretical foundations for modification of BFS and preparation of mineral wool (Gifford and Gillott 1996; Li et al. 2013).

## Methods

### Thermodynamic calculation with FactSage 6.4

Thermodynamic simulation was carried out by using FactSage [version 6.4, Thermfact/CRCT (Montreal) and GTT technologies (Aachen)], which provides theoretical support for the complex calculation and simulation of thermodynamic process. FactSage software was established based on the minimum Gibbs principle. Among the software, the “Eqilib” module (Li et al. 2013) can calculate all kinds of slag system to reach the equilibrium state at different temperature in different phases and quality. “Equilib” module was used to simulate the thermodynamic equilibrium, and the FToxide database was selected for this study. All calculations were carried out under an atmospheric pressure, and the experimental atmosphere was air. The temperature was selected from 900 to 1600 °C, and the increment was 5 °C (Cheng et al. 2013; Cao et al. 2008; Bale et al. 2002; Luz et al. 2011a, b).

### Hot remelting experimental and X-ray analysis

Remelting slag samples were prepared with analytical reagent of CaO, SiO_2_, Al_2_O_3_ and MgO, which were dried 6 h at 105 °C. A certain amounts of analytical reagents were weighted and mixed according to Table 1, and then 300 g remelting sample was transferred into a graphite crucible (outer diameter 80 mm, inner diameter 66 mm, height 100 mm). The crucibles were placed in a tube furnace and heated to melt, and all experiments were carried out under an atmospheric pressure, and the experimental atmosphere was air. The heating and cooling process was operated as the following: first stage, the heating rate of the furnace was set at 4 °C/min and lasted for 50 min to improve the temperature from room temperature to 200 °C. Second stage, the heating rate was set at 12 °C/min and lasted for 50 min to increase the temperature from 200 to 800 °C. Third stage, the heating rate was set at 10 °C/min and lasted for 40 min to improve the temperature from 800 to 1200 °C. Fourth stage, the heating rate was set at 6 °C/min and lasted for 50 min to improve the temperature from 1200 to 1500 °C. Fifth phase, the furnace temperature remained constant under 1500 °C for 120 min to remelt the sample completely. Sixth stage, the cooling rate was set at −5 °C/min and lasted for 50 min to decline the temperature from 1500 to 1250 °C. Seventh stage, the furnace temperature remained constant under 1250 °C for 30 min to guarantee thermodynamic equilibrium of the high melting mineral phases. Finally, the crucible was taken out and quickly immersed into water to quench the molten slag sample to reserve the equilibrating phases.

**Table 1:** Chemical component of the remelting samples (mass fraction, %)

No.	CaO	SiO_2_	Al_2_O_3_	MgO	R (CaO/SiO_2_)
1	39.00	39.00	13	9	1.0
2	40.86	37.14	13	9	1.1
3	42.55	35.45	13	9	1.2
4	44.09	37.14	13	9	1.3
5	40.50	40.50	10	9	1.0
6	37.50	37.50	16	9	1.0
7	40.50	40.50	13	6	1.0
8	37.50	37.50	13	12	1.0

For XRD analysis, the cooling samples were measured on a D/MAX2500PC X-ray diffractometer, used Cu-Kα radiation operated at 40 kV and 100 mA. All the diffraction profiles were obtained in continuous mode at the scan speed of 10°/min, and the 2θ range from 10° to 80°. Before analysis, the cooling samples were grinded and screened. The powder samples with particle size <0.048 mm were placed into the groove of glass sheets and smoothed. Then the glass sheets were put into the X-ray diffraction instrument (Ma 2010; Hu et al. 2004).

## Results and discussion

Based on original BFS and its modification target, the chemical compositions of the series of quaternary slag system selected in this research were listed in Table 1. As presented in Table 1, the samples from No. 1 to No. 4 could exhibit the variation of slag basicity due to their basicity being 1.0, 1.1, 1.2 and 1.3, respectively, whilst MgO and Al_2_O_3_ content were 9 and 13 %, respectively. The samples of No. 1, No. 5 and No. 6 could show the changing of Al_2_O_3_ content, because the Al_2_O_3_ content being 13, 10 and 16 %, respectively, whilst the basicity and MgO content were 1.0 and 9 %, respectively. For the samples of No. 1, No. 7, and No. 8, the basicity and MgO content were 1.0 and 13 %, respectively, whilst MgO content were 9, 6 and 12 %, which could applied to analyse the influence of MgO content on the crystallization of mineralogical phases.

### Influence of basicity on mineralogical phases

**Fig. 1:**
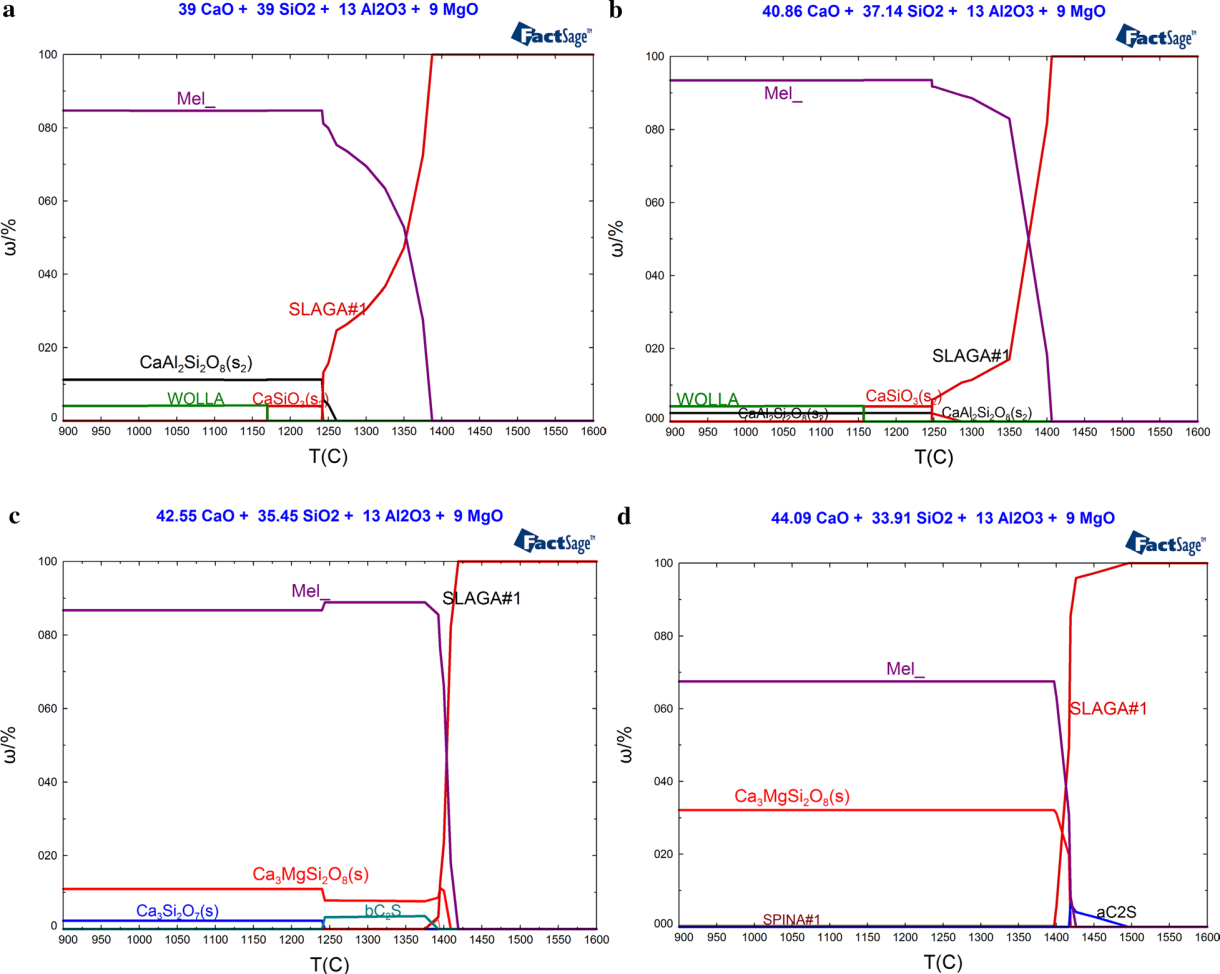
Changement of the mineralogical phases content along with decreasing of temperature under the condition of slag basicity being 1.0, 1.1, 1.2 and 1.3, respectively. **a** R = 1.0, **b** R = 1.1, **c** R = 1.2 and **d** R = 1.3

When Al_2_O_3_ and MgO content were 13 and 9 %, respectively, the mineralogical phases of slag with basicity of 1.0, 1.1, 1.2 and 1.3 under different temperature varied from 900 to 1600 °C with an increment of 5 °C have been calculated by FactSage 6.4 thermodynamic software. The changing laws of the mineralogical phases content along with temperature were illustrated in Fig. 1. As shown in Fig. 1a for the slag basicity is 1.0, the quantity of liquid phase (denoted with SLAG#1 in Fig. 1) decreased gradually with the reducing temperature. When the temperature declined to 1387.2 °C, the solid phase, namely melilite (denoted with Mel_ in Fig. 1), start to crystallize. As the temperature declined to 1260.7 °C, anorthite (denoted with CaAl_2_Si_2_O_8_ in Fig. 1) has been crystallized. At this point, the mass ratio (ω/%) of melilite crystallized in this system reached to 75.9 %. When the temperature declined to 1243.2 °C, the liquid melt has transformed into solid phase completely, and calcium metasilicate (denoted with CaSiO_3_ in Fig. 1) has been crystallized. At this temperature, the mass ratio of melilite and anorthite reached to 84.72 and 11.25 %, respectively, and keep constant with the further declining of temperature. Due to the instability, calcium metasilicate has transformed to wollastonite (denoted WOLLA in Fig. 1). As the temperature declined to 1169.2 °C, the mass ratio of wollastonite was 4.03 %.

As shown in Fig. 1b for the slag basicity of 1.1, the mineralogical phases composed of melilite, anorthite, calcium metasilicate and wollastonite, were the similar to those in the slag with basicity of 1.0 with the declining of temperature. The crystallization temperature of melilite and calcium metasilicate increased to 1406.3 and 1247.4 °C, respectively, while those of anorthite and transforming temperature of calcium metasilicate declined to 1286.8 and 1156.7 °C, respectively. Compared with the crystallization quantities of different mineralogical phases in Fig. 1a, the mass ratio of melilite increased to 93.43 %, while those of anorthite, calcium metasilicate and its transformation product, namely wollastonite, declined observably.

When the basicity of slag increased to 1.2 as shown in Fig. 1c, though the main mineralogical phases were melilite, magnesium rhodonite (denoted with Ca_3_MgSi_2_O_8_ in Fig. 1), dicalcium silicate (denoted with α-Ca_2_SiO_4_ in Fig. 1) has been emerged and took the place of anorthite and calcium metasilicate. The crystallization temperature of melilite increased to 1418.9 °C, while those of manganolite rhodonite and dicalcium silicate were 1408.9 and 1393.0 °C, respectively. Unstable dicalcium silicate was transformed to stable rankinite at the temperature of 1243.6 °C. When the temperature of slag declined to 1200 °C, the mass ratios of melilite and manganolite rhodonite were 87.82 and 10.06 %, respectively, while that of rankinite is only 2.12 %.

As shown in Fig. 1d with the slag basicity of 1.3, the crystallization phases were mainly melilite and magnesium rhodonite, which are similar to Fig. 1c. The crystallized content of melilite decreased, while that of magnesium rhodonite increased. Compared with the slag sample with the basicity of 1.2, the crystallized content of dicalcium silicate increased. Along with the increasing of basicity, the crystallization temperature of dicalcium silicate, melilite and magnesium rhodonite increased to 1495.6, 1425 and 1418.9 °C, respectively. When the temperature dropped to 1399.6 °C, a trace amount of spinel (denoted with SPINA in Fig. 1) was crystallized and its content is only 0.34 %, while the mass ratios of melilite and magnesium rhodonite were 67.46 and 32.2 %, respectively.

**Fig. 2:**
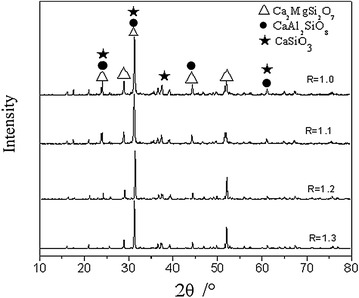
X-ray diffraction pattern of the remelting slag sample

The accuracy of the simulation results were verified by remelting experiment. Figure 2 shows the X-ray diffraction pattern of the remelting slag samples, which the basicity was 1.0, 1.1, 1.2 and 1.3, while Al_2_O_3_ and MgO content were 13 and 9 %, respectively. Those chemical compositions were identical to that of the sample corresponding to Fig. 1. As illustrated in Fig. 2, the mineral phases of the remelting slag sample whose basicity was 1.0 were composed of calcium magnesium melilite (denoted with CaMgSi_2_O_7_ in Fig. 2), anorthite (denoted with CaAl_2_Si_2_O_8_ in Fig. 2) and calcium metasilicate (denoted with CaSiO_3_ in Fig. 2). The mineral phases of the remelting slag sample in Fig. 2, whose basicity was 1.1 were the same as the basicity of 1.0. The mineral phases of the remelting slag sample whose basicity was 1.2 as illustrated in Fig. 2 were composed of calcium magnesium melilite (denoted with CaMgSi_2_O_7_ in Fig. 2) and dicalcium silicate (denoted with Ca_2_SiO_4_ in Fig. 2). As illustrated in Fig. 2, only calcium magnesium melilite (denoted with CaMgSi_2_O_7_ in Fig. 2) was found in the remelting slag sample whose basicity was 1.3. It was possible that the amount of SPINA was too low to be found. The results were similar to the mineralogical phases shown in Fig. 1 at the temperature of 1250 °C, and it was feasible to simulate the mineralogical composition of quaternary slag system by FactSage 6.4 thermodynamic software. Accordingly, the water quenching method could keep the mineralogical phases in the slag sample at high temperature, that is to say, the mineralogical composition obtained at room temperature from the sample undergone water quenching could reflect that at high temperature.

In general, the main mineralogical phase of this series of slag system was melilite. The crystallization temperature of melilite increased with the increasing of basicity, while the mass ratio of crystallization trends to increase to summit and then decrease. Meanwhile, basicity of the slag system has a significant influence on the type and crystallization temperature of the mineralogical phases. When the basicity of slag was 1.0, a certain amount of anorthite and wollastonite could be crystallized with the declining of temperature. The mass ratios of anorthite and wollastonite decreased gradually with the increasing of basicity. When the basicity of slag was increased to 1.2, anorthite and calcium metasilicate were gradually replaced by magnesium rhodonite and dicalcium silicate, respectively. When the basicity of slag increased to 1.3, the mass ratio of magnesium rhodonite crystallized increased significantly and finally there was a certain amount of α-Ca_2_SiO_4_. The changing law of the dicalcium silicate content with the basicity is consistent with the results of the Gelfi_M (Gelf et al. 2010), in which the influence of basicity on dicalcium silicate content and the leaching behavior of EAF steel slag were investigated.

### Influence of Al_2_O_3_ content on mineralogical phases

**Fig. 3:**
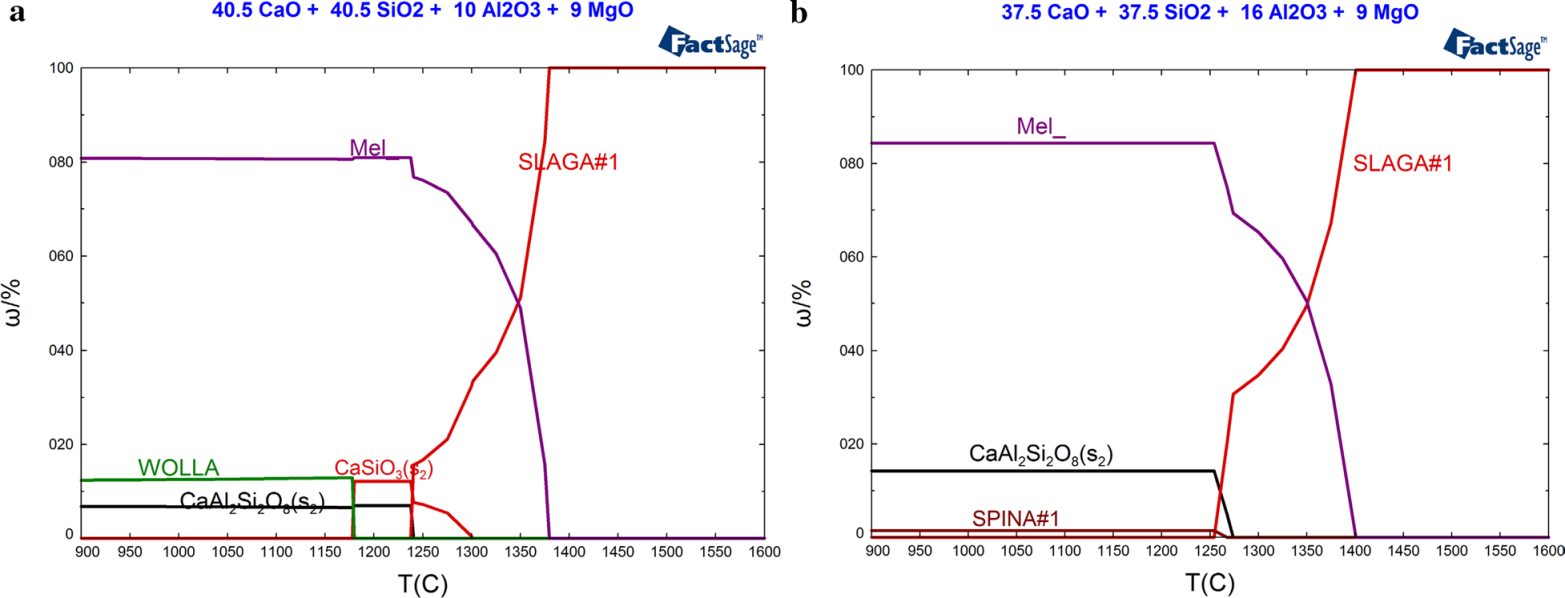
Changement of the mineralogical phases content along with decreasing of temperature under the condition of Al_2_O_3_ content being 10 and 16 %. **a** ω(Al_2_O_3_) = 10 %, **b** ω(Al_2_O_3_) = 16 %

When the basicity and MgO content were 1.0 and 9 %, respectively, the mineralogical phases of slag samples with Al_2_O_3_ content of 10 and 16 % under different temperature varied from 900 to 1600 °C with an increment of 5 °C have been calculated by FactSage 6.4 thermodynamic software. The changing laws of the mineralogical phases content along with temperature were illustrated in Fig. 3. Combined with Fig. 1a with Al_2_O_3_ content of 13 %, the influence of Al_2_O_3_ content on mineralogical phases of the slag system could be analyzed.

As shown in Fig. 3a corresponding to the slag sample with Al_2_O_3_ content being 10 %, the mineralogical phases crystallized during cooling process were identical to those of the slag sample with Al_2_O_3_ content being 13 % illustrated in Fig. 1a. As Al_2_O_3_ content was decreased from 13 to 10 %, the crystallization temperature of melilite and anorthite declined from 1387.2 and 1260.7 to 1379.5 and 1240.5 °C, respectively. When the temperature dropped to 1301.1 °C, calcium metasilicate started to crystallize from the slag system. At this temperature, the mass ratio of melilite crystallized reached to 66.57 %. As the temperature declined to 1240.5 °C, anorthite commenced to crystallize, and the mass ratios of melilite and calcium metasilicate crystallized have reached to 76.79 and 7.6 %, respectively. When the temperature declined to 1179.7 °C, the unstable compound named calcium metasilicate has transferred into wollastonite, which content was 7.6 %. At this temperature, the mass ratios of melilite and anorthite crystallized were 80.93 and 6.56 %, respectively.

Compared Fig. 3b and Fig. 1a with Al_2_O_3_ content of 16 and 13 %, respectively, the main mineralogical phases were melilite and anorthite. As shown in Fig. 3b, the crystallization temperature of slag sample increased to 1400.7 and 1273.7 °C, as Al_2_O_3_ content increased to 16 %. Meanwhile, calcium metasilicate could not be crystallized and replaced by spinel which crystallized at 1267.4 °C. At 1250 °C, the mass ratios of melilite and anorthite crystallized were about 84 and 14.27 %, respectively, while that of spinel was only 1.43 %.

**Fig. 4:**
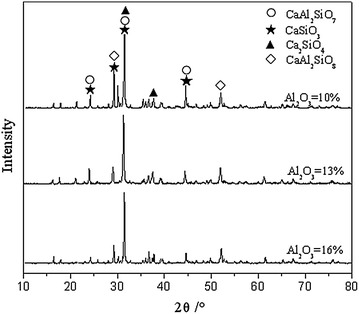
X-ray diffraction pattern of the remelting slag sample

The accuracy of the simulation results were verified by remelting experiment. Figure 4 shows the X-ray diffraction pattern of the remelting slag sample with Al_2_O_3_ content of 10 and 16 %, while the basicity and MgO content were 1.0 and 9 %, respectively, which chemical composition was identical to that of the sample corresponding to Fig. 3. As illustrated in the Fig. 4, the main mineralogical phases of the remelting slag sample whose Al_2_O_3_ was 10 % were composed of aluminum melilite (denoted with Ca_2_Al_2_SiO_7_ in Fig. 4), anorthite and calcium metasilicate. As illustrated in the Fig. 4, aluminum melilite (denoted with Ca_2_Al_2_SiO_7_ in Fig. 4) and spinel were be found in the remelting slag sample whose Al_2_O_3_ was 16 %. The results were similar to the mineralogical phases shown in Fig. 3 at the temperature of 1250 °C. In addition, due to the remelting slag samples were prepared with chemical pure reagents and the heterogeneity of the remelting slag sample during mixing, remelting and cooling process, there was a trace amount of dicalcium silicate. A little of dicalcium silicate crystallized in the remelting slag sample has little effect on the result and the mineralogical composition was consistent with that simulated with thermodynamics software.

In general, the main mineralogical phases of the slag systems were melilite and anorthite, which crystallization temperature could be improved along with the increasing of Al_2_O_3_ content. Furthermore, there was a tendency that the mass ratios of melilite and anorthite crystallized increased with Al_2_O_3_ content. When the Al_2_O_3_ content were 10 and 13 % (shown in Figs. 1a, 3a), a certain amount of calcium metasilicate could be crystallized. The mass ratio of calcium metasilicate crystallized trend to decrease gradually with the increasing of Al_2_O_3_ content. When Al_2_O_3_ content increased to 16 % (shown in Fig. 3b), calcium metasilicate could not be crystallized and substituted by spinel.

### Influence of MgO content on mineralogical phases

**Fig. 5:**
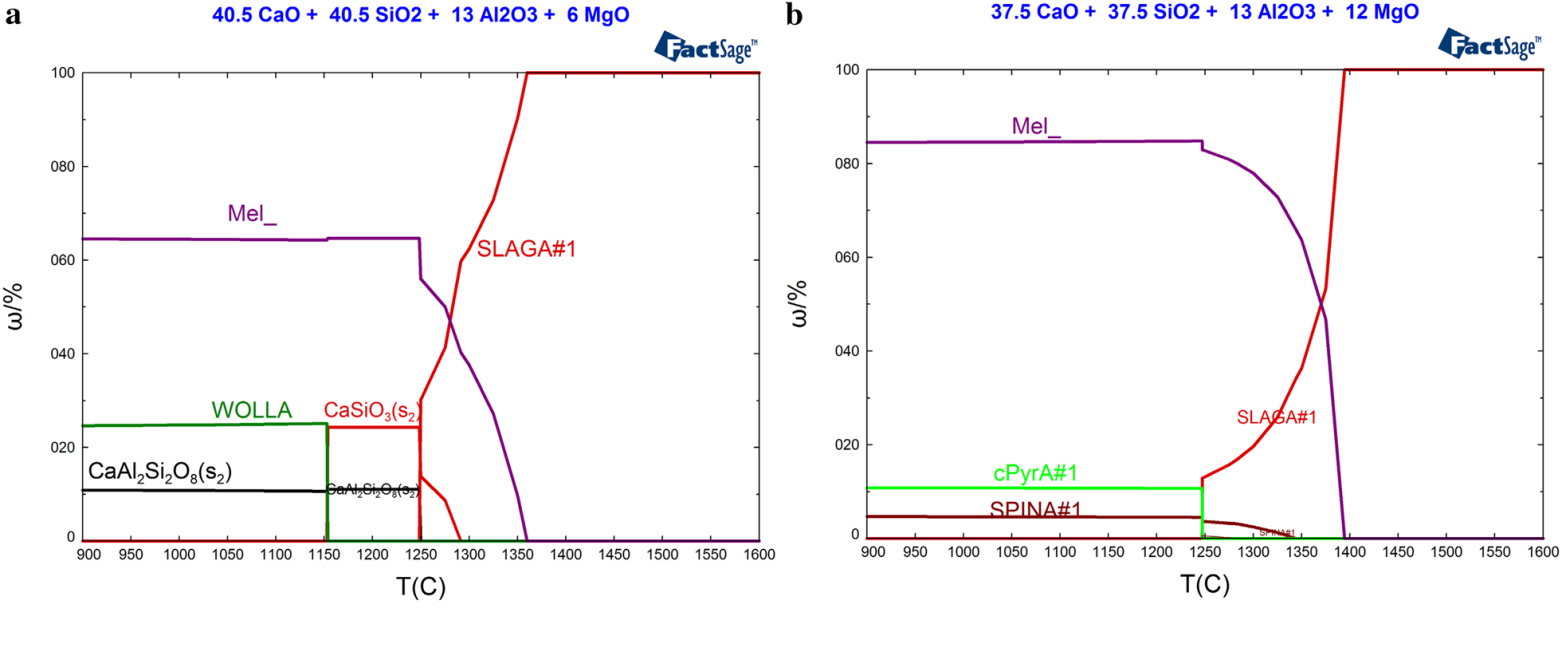
Changement of the mineralogical phases content along with decreasing of temperature under the condition of MgO content being 6 and 12 %. **a** ω(MgO) = 6 %, **b** ω(MgO) = 12 %

When the basicity and Al_2_O_3_ content were 1.0 and 13 %, respectively, the mineralogical phases of slag with MgO content of 6 and 12 % under different temperature varied from 900 to 1600 °C with an increment of 5 °C have been calculated by FactSage 6.4 thermodynamic software. The changing laws of the mineralogical phases content along with temperature were illustrated in Fig. 5. Combined with Fig. 1a with MgO content of 9 %, the influence of MgO content on mineralogical phases of the slag system could be analyzed.

As shown in Fig. 5a, the mineralogical composition of the slag sample with MgO content of 6 % was identical to that the slag sample with MgO content of 9 % illustrated in Fig. 1a. When MgO content decreased from 9 to 6 %, the crystallization temperature of melilite and anorthite declined from 1387.2 and 1260.7 to 1359.7 and 1249.7 °C, respectively, while that of calcium metasilicate increased from 1243.2 to 1291.2 °C. It was showed that the mass ratio of melilite was 40.29 % at the temperature of calcium metasilicate crystallization, which was much less than crystallization amount as MgO content was 9 %. When the temperature declined to the point of anorthite crystallization, the mass ratios of melilite and calcium metasilicate crystallized were 56.02 and 7.6 %, respectively. At 1179.7 °C, unstable calcium metasilicate should transform the species of wollastonite, and the mass ratio of melilite, anorthite and wollastonite were 24.61, 64.67 and 11.04 %, respectively.

Comparing the slag sample with MgO content of 6 and 12 % shown in Fig. 5a, b, respectively, the main mineralogical phase was melilite although MgO content increased to 12 % shown in Fig. 5b. It was concluded that calcium metasilicate could not be crystallized or replaced by spinel as MgO content increased. Meanwhile, anorthite was disappearred and substituted by pyroxene (denoted with cPyrA in Fig. 5). The crystallization temperature of melilite, spinel and pyroxene were 1392.3, 1344 and 1247 °C, respectively. At 1247 °C, the mass ratios of melilite, spinel and pyroxene crystallized were 84.46, 4.46 and 10.81 %.

**Fig. 6:**
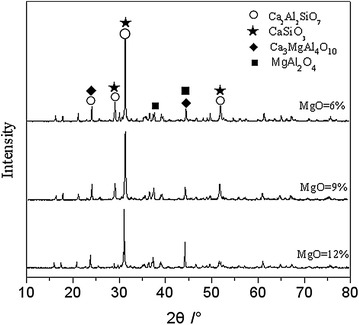
X-ray diffraction pattern of the remelting slag

The accuracy of the simulation results were verified by remelting experiment. Figure 6 shows the X-ray diffraction pattern of the remelting slag sample with MgO content of 6 and 12 %, while the basicity and Al_2_O_3_ were 1.0 and 13 %, respectively, whose chemical composition was similar to the sample corresponding to Fig. 5. As illustrated in the Fig. 4, the main mineralogical phases of the remelting slag sample whose MgO was 6 % were composed of aluminum melilite (denoted with Ca_2_Al_2_SiO_7_ in Fig. 6), calcium metasilicate and periclase (denoted with Ca_3_MgAl_4_O_10_ in Fig. 6). As illustrated in Fig. 4, spinel (denoted with MgAl_4_O_10_ in Fig. 6) was found in the remelting slag sample whose MgO was 12 %. The results were similar to the mineralogical phases shown in Fig. 5 at the temperature of 1250 °C. Although anorthite was replaced by periclase, as mentioned earlier, the heterogeneity of the remelting slag sample during mixing, remelting and cooling process maybe the reason to bring about this phenomena.

In general, the main mineralogical phase of the slag system was melilite. Both crystallization temperature and mass ratio of melilite increased with the increasing MgO content. When MgO content was 6 %, a certain amounts of anorthite, calcium metasilicate and wollastonite should be crystallized except melilite. With the increasing of MgO content, the mass ratio of anorthite, calcium metasilicate and wollastonite crystallized declined. Finally, anorthite and calcium metasilicate were replaced by pyroxene and spinel, respectively, as MgO content increased to 12 %.

## Conclusion

It is necessary to elucidate the crystallization thermodynamic of mineralogical phases in the melt BFS intent to fiberization, because crystalline phases have an important influence on application performance of the mineral wool. Therefore, the crystallization thermodynamic of the quaternary slag system of CaO–SiO_2_–Al_2_O_3_–MgO has been simulated with thermodynamics software FactSage 6.4 in the present study. Simultaneously, the hot remelting test and X-ray analysis have been carried out to determine the mineralogical composition of this quaternary slag system under the temperature of 1250 °C. It was shown that the results of X-ray analysis were consistent with that simulated with FactSage 6.4. In conclusion, it is feasible to keep the mineralogical phases in the slag sample at high temperature with the cooling method of water quenching, and to simulate the mineralogical composition of the quaternary slag system by FactSage 6.4.

Temperature and chemical composition, especially basicity, MgO and Al_2_O_3_ content, have significant influence on crystallization and transformation process of the high-melting point mineralogical phases. When the basicity, Al_2_O_3_ and MgO content were 1.0, 13 and 9 %, respectively, the crystallizing phases were composed of melilite, anorthite and calcium metasilicate. The mass ratio of melilite crystallized trend to increase to summit and then declined, while that of calcium metasilicate declined gradually with the increasing of the basicity. As the basicity increased to 1.2, anorthite was replaced by magnesium rhodonite. When the basicity and MgO content were 1.0 and 9 %, the mass ratio of melilite and anorthite crystallized increased, while that of calcium silicate declined, and replaced by spinel finally, with the increasing of Al_2_O_3_ content. When the basicity and Al_2_O_3_ content were 1.0 and 13 %, the mass ratio of melilite crystallized increased, while those of anorthite and calcium silicate declined, and replaced by pyroxene and spinel with the increasing of MgO content.

The fiberized temperature is a key parameter for production of the minerla wool, because the high-melting point mineralogical phases such as melilite, dicalcium silicate, anorthite, etc. could be crystallized with the declining of temperature. According to the crystallization thermodynamic results calculated with FactSage 6.4, the crystallization temperature of the primary crystallize phase such as melilite, anorthite, etc. increased with the increasing of the basicity, MgO and Al_2_O_3_ content in the slag system. Consequently, to decline the fiberization temperature of the melt BFS, the basicity, MgO and Al_2_O_3_ content should be decreased during the modification process of chemical composition.
